# Supplementary material for: Importance of Completing Hybrid Cardiac Rehabilitation for Long-Term Outcomes: A Real-World Evaluation
Source: J Clin Med. 2019 Feb 28;8(3):290. doi: 10.3390/jcm8030290 (PMC6462989; doi:10.3390/jcm8030290)
Supplement: Supplementary file 1 [file jcm-08-00290-s001.pdf]

Supplementary Material, Suskin et al, Importance of completing hybrid cardiac rehabilitation for long-term outcomes: A real-world evaluation

Supplement Figure: Outcomes - secondary analysis: Restrict matched pairs to those where both patients survived at least 1 year (365 days) following index date and were event free in the 365 days.
Supplement Table 1. The RECORD Checklist of Items That Should Be Reported in Observational Studies Using Routinely Collected Health Data
Supplement Table 2. Data Sources Used in the Study
Supplement Table 3. Patient CR Eligible Diagnoses & Source
Supplement Table 4 Administrative Data Codes Used to Define Baseline Characteristics & Source
Supplement Table 5. Administrative data codes used to define outcomes
Supplement Table 6. Pre-match Baseline Patient Characteristics

Supplement: Figure Kaplan–Meier survival curve * for secondary analysis **

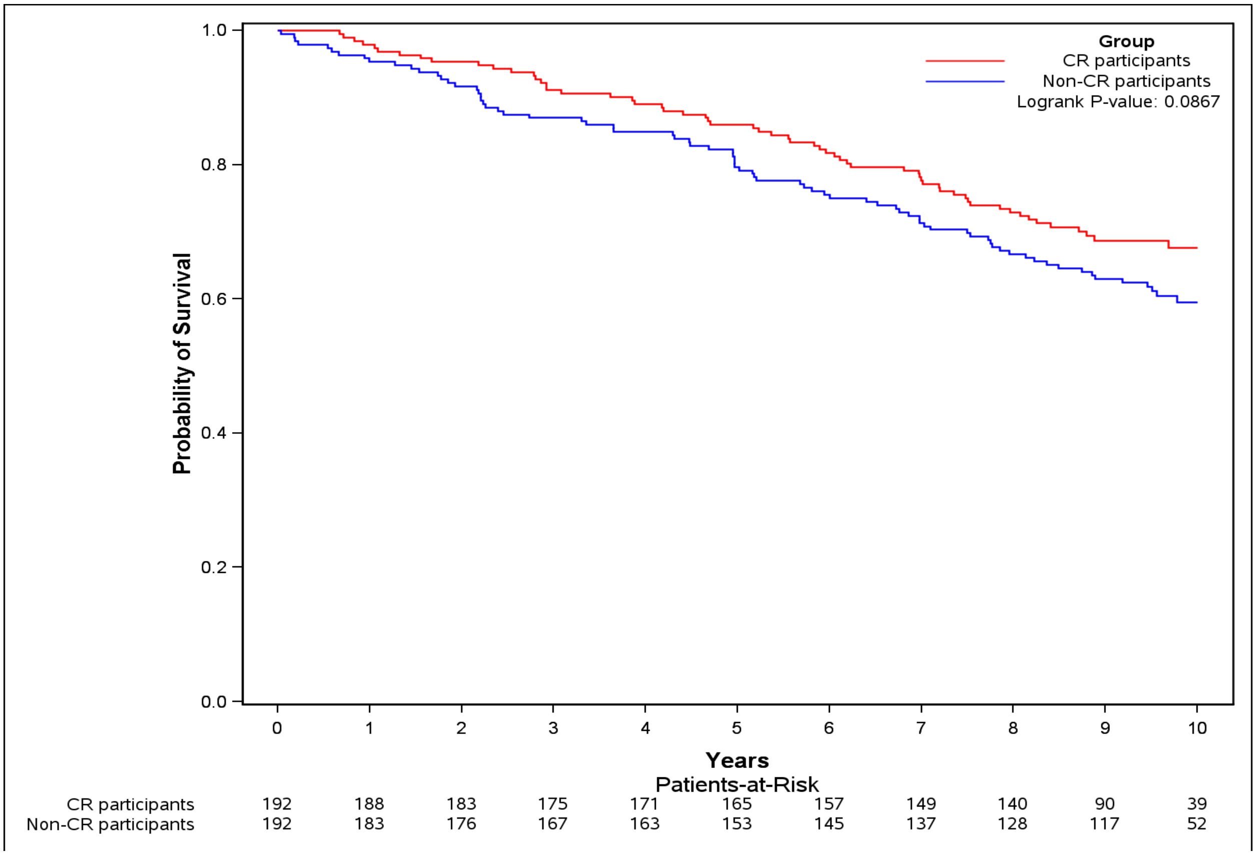

* The Kaplan Meier curve of the primary outcome (death or hospitalization for MI, HF, PCI, or CABG) was plotted and logrank test was performed.
** The secondary analysis assessed for the composite outcome of death, or re-hospitalization for MI, or PCI, or CABG, or HF during follow-up restricting the sample to those pairs who were event-free at 1-year after index date (CR entry for CR participant or matched date for non-CR participant)

Supplement Table 1. The RECORD Checklist of Items That Should Be Reported in Observational Studies Using Routinely Collected Health Data [1] [2]

		STROBE items	Location in manuscript where items are reported	RECORD items	Location in manuscript where items are reported
**Title and abstract**					
	1	(a) Indicate the study's design with a commonly used term in the title or the abstract (b) Provide in the abstract an informative and balanced summary of what was done and what was found	Abstract	RECORD 1.1: The type of data used should be specified in the title or abstract. When possible, the name of the databases used should be included.<br><br>RECORD 1.2: If applicable, the geographic region and timeframe within which the study took place should be reported in the title or abstract.<br><br>RECORD 1.3: If linkage between databases was conducted for the study, this should be clearly stated in the title or abstract.	Abstract (specific databases are described in the Methods and in Supple-ment Tables)<br><br>Linkage described in the Methods
**Introduction**					
Background rationale	2	Explain the scientific background and rationale for the investigation being reported	Introduction		
Objectives	3	State specific objectives, including any prespecified hypotheses	Introduction		
**Methods**					
Study Design	4	Present key elements of study	Methods		

[1] Benchimol EI, Smeeth L, Guttmann A, Harron K, Moher D, Petersen I, Sørensen HT, von Elm E, Langan SM, the RECORD Working Committee. The REporting of studies Conducted using Observational Routinely-collected health Data (RECORD) Statement. PLoS Medicine 2015; in press.

[2] Checklist is protected under Creative Commons Attribution (CC BY) license.

		design early in the paper			
Setting	5	Describe the setting, locations, and relevant dates, including periods of recruitment, exposure, follow-up, and data collection	Methods		
Participants	6	*(a) Cohort study* - Give the eligibility criteria, and the sources and methods of selection of participants. Describe methods of follow-up<br>*Case-control study* - Give the eligibility criteria, and the sources and methods of case ascertainment and control selection. Give the rationale for the choice of cases and controls<br>*Cross-sectional study* - Give the eligibility criteria, and the sources and methods of selection of participants<br><br>*(b) Cohort study* - For matched studies, give matching criteria and number of exposed and unexposed<br>*Case-control study* - For matched studies, give matching criteria and the number of controls per case	Methods	RECORD 6.1: The methods of study population selection (such as codes or algorithms used to identify subjects) should be listed in detail. If this is not possible, an explanation should be provided.<br><br>RECORD 6.2: Any validation studies of the codes or algorithms used to select the population should be referenced. If validation was conducted for this study and not published elsewhere, detailed methods and results should be provided.<br><br>RECORD 6.3: If the study involved linkage of databases, consider use of a flow diagram or other graphical display to demonstrate the data linkage process, including the number of individuals with linked data at each stage.	Methods; (Supple-ment Tables)<br><br>Methods (Figure)

Variables	7	Clearly define all outcomes, exposures, predictors, potential confounders, and effect modifiers. Give diagnostic criteria, if applicable.	Methods	RECORD 7.1: A complete list of codes and algorithms used to classify exposures, outcomes, confounders, and effect modifiers should be provided. If these cannot be reported, an explanation should be provided.	Supple-ment (Tables)
Data sources/ measurement	8	For each variable of interest, give sources of data and details of methods of assessment (measurement). Describe comparability of assessment methods if there is more than one group	Methods		
Bias	9	Describe any efforts to address potential sources of bias	Methods		
Study size	10	Explain how the study size was arrived at	Methods; Figure		
Quantitative variables	11	Explain how quantitative variables were handled in the analyses. If applicable, describe which groupings were chosen, and why	Methods		
Statistical methods	12	(a) Describe all statistical methods, including those used to control for confounding<br>(b) Describe any methods used to examine subgroups and interactions	(a) Methods<br>(b) Methods<br>(c) n/a		

		(c) Explain how missing data were addressed<br>(d) *Cohort study* - If applicable, explain how loss to follow-up was addressed<br>*Case-control study* - If applicable, explain how matching of cases and controls was addressed<br>*Cross-sectional study* - If applicable, describe analytical methods taking account of sampling strategy<br>(e) Describe any sensitivity analyses	(d) Methods<br>(e) Methods (secondary and) sensitivity analyses		
Data access and cleaning methods		..		RECORD 12.1: Authors should describe the extent to which the investigators had access to the database population used to create the study population.<br><br>RECORD 12.2: Authors should provide information on the data cleaning methods used in the study.	Methods
Linkage		..		RECORD 12.3: State whether the study included person-level, institutional-level, or other data linkage across two or more databases. The methods of linkage and methods of linkage quality evaluation should be provided.	Methods
**Results**					

Participants	13	(a) Report the numbers of individuals at each stage of the study (*e.g.*, numbers potentially eligible, examined for eligibility, confirmed eligible, included in the study, completing follow-up, and analysed)<br>(b) Give reasons for non-participation at each stage.<br>(c) Consider use of a flow diagram	(a) Methods & Results (Tables & Figures)<br>(b) Results (Figures)<br>(c) Results (Figures)	RECORD 13.1: Describe in detail the selection of the persons included in the study (*i.e.,* study population selection) including filtering based on data quality, data availability and linkage. The selection of included persons can be described in the text and/or by means of the study flow diagram.	Results (Tables & Figures)
Descriptive data	14	(a) Give characteristics of study participants (*e.g.*, demographic, clinical, social) and information on exposures and potential confounders<br>(b) Indicate the number of participants with missing data for each variable of interest<br>(c) *Cohort study* - summarise follow-up time (*e.g.*, average and total amount)	(a) Results<br>(b) n/a<br>(c) Results		
Outcome data	15	*Cohort study* - Report numbers of outcome events or summary measures over time<br>*Case-control study* - Report numbers in each exposure category, or	Results (Tables and Figures)		

		summary measures of exposure *Cross-sectional study* - Report numbers of outcome events or summary measures			
Main results	16	(a) Give unadjusted estimates and, if applicable, confounder-adjusted estimates and their precision (e.g., 95% confidence interval). Make clear which confounders were adjusted for and why they were included (b) Report category boundaries when continuous variables were categorized (c) If relevant, consider translating estimates of relative risk into absolute risk for a meaningful time period	(a) Results (b) n/a (c) Results		
Other analyses	17	Report other analyses done—e.g., analyses of subgroups and interactions, and sensitivity analyses	Results (Tables & Figures)		
**Discussion**					
Key results	18	Summarise key results with reference to study objectives	Discussion		
Limitations	19	Discuss limitations of the study, taking into account sources of potential bias or imprecision. Discuss both direction and magnitude of any potential bias	Limitations	RECORD 19.1: Discuss the implications of using data that were not created or collected to answer the specific research question(s). Include discussion of misclassification bias,	Methods & Limitations

				unmeasured confounding, missing data, and changing eligibility over time, as they pertain to the study being reported.	
Interpretation	20	Give a cautious overall interpretation of results considering objectives, limitations, multiplicity of analyses, results from similar studies, and other relevant evidence	Discussion		
Generaliz-ability	21	Discuss the generalizability (external validity) of the study results	Discussion & Limitations		
**Other Information**					
Funding	22	Give the source of funding and the role of the funders for the present study and, if applicable, for the original study on which the present article is based	Acknowledge-ments		
Accessibility of protocol, raw data, and programming code				RECORD 22.1: Authors should provide information on how to access any supplemental information such as the study protocol, raw data, or programming code.	Data Sharing Agreement [3]

[3] The dataset from this study is held securely in coded form at ICES. While data sharing agreements prohibit ICES from making the dataset publicly available, access may be granted to those who meet pre-specified criteria for confidential access, available at www.ices.on.ca/DAS. The full dataset creation plan and underlying analytic code are available from the authors upon request, understanding that the computer programs may rely upon coding templates or macros that are unique to ICES and are therefore either inaccessible or may require modification

Supplement Table 2. Data Sources Used in the Study

**Database**	**Description**
Health Services	
Discharge Abstract Database (DAD)	The DAD is compiled by the Canadian Institute for Health Information (CIHI) and contains administrative, clinical (diagnoses and procedures/interventions), demographic, and administrative information for all admissions to acute care hospitals in Ontario. At ICES, consecutive DAD records are linked together to form 'episodes of care' among the hospitals to which patients have been transferred after their initial admission. Prior to April 1, 2002, diagnoses (up to 16 on a given DAD record) are captured using the International Statistical Classification of Diseases, Injuries, and Causes of Death, 9th Revision (ICD-9) coding system and procedures (up to 10 on a given DAD record) are captured using the Canadian Classification of Diagnostic, Therapeutic, and Surgical Procedures (CCP) coding system. Following April 1, 2002, diagnoses (up to 25 on a given DAD record) are captured using the International Statistical Classification of Diseases and Related Health Problems, 10th Revision, Canada (ICD-10-CA) coding system and interventions (up to 20 on a given DAD record) are captured using the Canadian Classification of Health Interventions (CCI) coding system. In a hospital medical record re-abstraction study of 14,500 hospital discharges from 18 hospital sites between April 2002 and March 2004, DAD records were demonstrated to have excellent agreement (over 99%) for nonmedical information such as demographic and administrative data. Regarding diagnoses, median agreement between the original DAD records and the reabstracted records for the 50 most common most responsible diagnoses was 81% (Sensitivity 82%; Specificity 82%).(2) The corresponding median agreement for the 50 most frequently performed surgical procedures was 92% (sensitivity 95%, positive predictive value 91%).

National Ambulatory Care Reporting System (NACRS)	The NACRS is compiled by the Canadian Institute for Health Information (CIHI) and contains administrative, clinical (diagnoses and procedures), demographic, and administrative information for all patient visits made to hospital- and community- based ambulatory care centres (emergency departments, day surgery units, hemodialysis units, and cancer care clinics) in Ontario. At ICES, NACRS records are linked with other data sources (DAD, Ontario Mental Health Reporting System [OMHRS]) to identify transitions to other care settings, such as inpatient acute care or psychiatric care.<br><br>Prior to April 1, 2002, diagnoses (up to 6 on a given NACRS record) are captured using the ICD-9 coding system and procedures (up to 10 on a given NACRS record) are captured using the CCP coding system. Following April 1, 2002, diagnoses (up to 10 on a given NACRS record) are captured using the ICD- 10-CA coding system and interventions (up to 10 on a given NACRS record) are captured using the CCI coding system.<br>NACRS emergency department diagnosis codes have been extensively validated.
Ontario Health Insurance Plan (OHIP) Claims History Database	The OHIP claims database contains information on inpatient and outpatient services provided to Ontario residents eligible for the province's publicly funded health insurance system by fee-for- service health care practitioners (primarily physicians) and "shadow billings" for those paid through non-fee-for-service payment plans.<br><br>Billing codes on the claims (OHIP fee codes) identify the care provider, their area of specialization and the type and location of service. OHIP billing claims also contain a 3-digit diagnosis code - the main reason for the service - captured using a modified version of the ICD, 8th revision coding system. OHIP claims are well completed, but the validity of the diagnosis coding is highly variable.(4)
Same-Day Surgery (SDS) database	The SDS is compiled by the Canadian Institute for Health Information (CIHI) and contains administrative, clinical (diagnoses and procedures), demographic, and administrative information for all patient visits made to day surgery institutions in Ontario.<br><br>Prior to April 1, 2002, diagnoses (up to 16 on a given SDS record) were captured using the ICD-9 coding system and procedures (up to 10 on a given SDS record) were captured using the CCP coding system. Since April 1, 2002, diagnoses (up to 25 on a given SDS record) are captured using the ICD-

	10-CA coding system and interventions (up to 16 on a given SDS record) are captured using the CCI coding system.
ICES-derived cohorts	
Ontario Congestive Heart Failure (CHF) Database	The Ontario CHF Database is created using a definition of ≥2 physician billing claims with a diagnosis of CHF (OHIP diagnosis code: 428) and/or ≥1 inpatient hospitalization or same day surgery record with a diagnosis of CHF (ICD-9 diagnosis code: 428; ICD-10 diagnosis code: I50; in the primary diagnostic code space) in a two-year period applied to hospitalization (DAD), same day surgery (SDS), and physician billing claims (OHIP) data to determine the diagnosis date for incident cases of CHF in Ontario.<br><br>When using electronic medical record data abstraction as the reference standard, the above definition has been demonstrated to have the following performance characteristics: Sensitivity (84.8%), Specificity (97.0%), and Positive Predictive Value (55.3%).(6)
Ontario Chronic Obstructive Pulmonary Disease (COPD) Database	The Ontario COPD Database is created using two separate algorithms applied to inpatient hospitalization (DAD), same day surgery (SDS) records, and physician billing claims (OHIP) data to determine the diagnosis date for incident cases of COPD in Ontario.<br><br>In an algorithm which maximizes sensitivity, the definition for COPD is any physician billing claim with a diagnosis for COPD (OHIP diagnosis codes: 491, 492, 496) or any inpatient hospitalization or same day surgery record with a diagnosis for COPD (ICD-9 diagnosis codes: 491, 492, 496; ICD-10 diagnosis codes: J41- J44; in any diagnostic code space). When using expert panel review of primary care charts as the reference standard, this definition has been shown to have the following performance characteristics: Sensitivity (85.0%), Specificity (78.4%), Positive Predictive Value (57.5%), and Negative Predictive Value (93.8%).(7)<br><br>In an algorithm which maximizes specificity, the definition for COPD is ≥3 physician billing claims with a diagnosis for COPD (OHIP diagnosis codes: 491, 492, 496) or ≥1 inpatient hospitalization or same day surgery record with a diagnosis for COPD (ICD-9 diagnosis codes: 491, 492, 496; ICD-10 diagnosis codes: J41, J42, J43, J44; in any diagnostic code space) in a two- year period. When using expert panel review of primary care charts as the reference standard, this definition has been shown to have the following

	performance characteristics: Sensitivity (57.5%), Specificity (95.4%), Positive Predictive Value (81.3%), and Negative Predictive Value (86.7%).(7)
Ontario Diabetes Database (ODD)	The ODD is created using algorithms applied to inpatient hospitalization (DAD) records, same day surgery (SDS) records, and physician billing claims (OHIP) data to determine the diagnosis date for incident cases of diabetes in Ontario.<br><br>For adults aged 19 years and greater, the definition for diabetes is 2 physician billing claims with a diagnosis for diabetes (OHIP diagnosis code: 250) or 1 inpatient hospitalization or same day surgery record with a diagnosis for diabetes (ICD-9 diagnosis code: 250; ICD-10 diagnosis codes: E10, E11, E13, E14; in any diagnostic code space) within a 2 year period. Physician claims and hospitalizations with a diagnosis of diabetes occurring within 120 prior to and 180 days after a gestational hospitalization record were excluded. When using primary care chart abstraction as the reference standard, this definition has been shown to have the following performance characteristics: Sensitivity (86.1%), Specificity (97.1%), Positive Predictive Value (79.8%), and Negative Predictive Value (98.1%).(8) For individuals aged 18 years or less, the definition for diabetes is 4 physician billing claims with a diagnosis of diabetes (OHIP diagnosis code: 250) within a 2 year period. Physician claims during the newborn hospitalization episode were excluded. When using primary care chart abstraction as the reference standard, this definition has been shown to have the following performance characteristics: Sensitivity (82.8%), Specificity (98.9%), Positive Predictive Value (99.4%), and Negative Predictive Value (71.2%).(9)

Ontario Hypertension Database	The Ontario Hypertension Database is created using a definition of ≥2 physician billing claims with a diagnosis of hypertension (OHIP diagnosis codes: 401-405) and/or ≥1 inpatient hospitalization or same day surgery record with a diagnosis of hypertension (ICD-9 diagnosis codes: 401-405; ICD-10 diagnosis codes: I10-I13, I15; in any diagnostic code space) in a two-year period applied to hospitalization (DAD), same day surgery (SDS), and physician billing claims (OHIP) data to determine the diagnosis date for incident cases of hypertension in Ontario.<br>Physician claims and hospitalizations with a diagnosis of hypertension occurring within 120 prior to and 180 days after a gestational hospitalization record are excluded.<br><br>When using electronic medical record data abstraction as the reference standard, the above definition has been demonstrated to have the following performance characteristics: Sensitivity (72%), Specificity (95%), Positive Predictive Value (87%), and Negative Predictive Value (88%).(11)
Ontario Myocardial Infarction Database (OMID)	The OMID contains records of all inpatient hospital admissions for acute myocardial infarctions (ICD-9 diagnosis code: 410; ICD- 10 diagnosis code: I21; in the primary diagnostic code space) in Ontario since 1991. These admissions are ascertained using the DAD and exclude in-hospital events and admissions where there had been a previous discharge for acute myocardial infarction in the previous year. This cohort of patients with acute myocardial infarction hospital admissions is linked with hospitalization (DAD), same day surgery (SDS), and physician billing claims data (OHIP) to create indicators of hospital readmission after discharge and receipt of cardiac procedures during and after the initial hospital admission.<br><br>When using a clinical registry of acute coronary syndromes from 58 cardiac care units in Ontario as the reference standard, the above definition has been demonstrated to have the following performance characteristics: Sensitivity (92.8%), Specificity (88.9%), and Positive Predictive Value (88.5%).(12)
Acquired cohorts and registries	

Ontario Cancer Registry (OCR)	The OCR is a computerized database of information on all Ontario residents who have been newly diagnosed with cancer since 1964. All new cases of cancer, expect non-melanoma skin cancer, are registered in the information system which is managed and maintained by Cancer Care Ontario (CCO). Data from multiple sources, including DAD and SDS records from CIHI which include a diagnosis of cancer, paper reports from pathology departments with any mention of cancer, electronic reports from the eight Ontario Regional Cancer Centers and from the Princess Margaret Hospital (the specialized institutions treated cancer patients in Ontario), and electronic reports of all deaths of Ontario residents from the Office of the Registrar General of Ontario based on Ontario Provincial death certificates with cancer as the underlying cause of death are linked to compile incident cases of cancer in Ontario.<br><br>Approximately 95% of all diagnosed cancer cases in Ontario are captured by the OCR.(15) When using a clinical registry of head and neck tumours from a provincial regional cancer centre as the reference standard, there was excellent agreement with the OCR for tumour site (81%) and diagnosis date within 1 month (91.5%).(16)
Care provider and facility data	
ICES Physician Database (IPDB)	The IPDB provides information about all physicians who have practiced in Ontario and is comprised of data contained in the OHIP Claims History Database, the OHIP Corporate Provider Database (CPDB), and the Ontario Physician Human Resource Data Centre (OPHRDC) Database. The database contains information on demographics (age, gender, year of graduation, school of graduation); specialty (functional and certified); location of practice; and measures of physician activity (billings and workload data).
Population and demographics	
Office of the Registrar General (ORGD) Vital Statistics Database	The ORGD Vital Statistics Database contains information on all deaths registered in Ontario starting on January 1, 1990. Information on the causes of death (immediate, antecedent, and underlying) recorded on the death certificate are captured. At ICES, we derive a single cause of death variable based on the underlying cause of death if available and, otherwise, the immediate cause of death using the ICD-9 coding system.

OHIP Registered Persons Database (RPDB)	The OHIP RPDB provides basic demographic information (age, sex, location of residence, date of birth, and date of death for deceased individuals) for those issued an Ontario health insurance number. The RPDB also indicates the time periods for which an individual was eligible to receive publicly funded health insurance benefits and the best known postal code for each registrant on July 1st of each year.

Reference List

(1) Hirdes JP, Ljunggren G, Morris JN, Frijters DH, Finne SH, Gray L et al. Reliability of the interRAI suite of assessment instruments: a 12-country study of an integrated health information system. BMC Health Serv Res 2008; 8:277.

(2) Juurlink D, Preyra C, Croxford R, Chong A, Austin P, Tu J et al. Canadian Institute for Health Information Discharge Abstract Database: A Validation Study. 2006. Toronto, Institute for Clinical Evaluative Sciences.

(3) Levy AR, O'Brien BJ, Sellors C, Grootendorst P, Willison D. Coding accuracy of administrative drug claims in the Ontario Drug Benefit database. Can J Clin Pharmacol 2003;10(2):67-71.

(4) Williams J, Young W. A summary of the quality of health care administrative databases in Canada. In: Goel V, Williams J, Anderson G, Blackstien-Hirsch P, Fooks C, Naylor C, editors. Patterns of Health Care in Ontario: The ICES Practice Atlas. 2nd Edition. Ottawa: Canadian Medical Association; 1996. 339-346.

(5) Gershon AS, Wang C, Guan J, Vasilevska-Ristovska J, Cicutto L, To T. Identifying patients with physician-diagnosed asthma in health administrative databases. Can Respir J 2009; 16(6):183- 188.

(6) Schultz SE, Rothwell DM, Chen Z, Tu K. Identifying cases of congestive heart failure from administrative data: a validation study using primary care patient records. Chronic Dis Inj Can 2013; 33(3):160-166.

(7) Gershon AS, Wang C, Guan J, Vasilevska-Ristovska J, Cicutto L, To T. Identifying individuals with physcian diagnosed COPD in health administrative databases. COPD 2009; 6(5):388-394.

(8) Hux JE, Ivis F, Flintoft V, Bica A. Diabetes in Ontario: determination of prevalence and incidence using a validated administrative data algorithm. Diabetes Care 2002; 25(3):512-516.

(9) Guttmann A, Nakhla M, Henderson M, To T, Daneman D, Cauch-Dudek K et al. Validation of a health administrative data algorithm for assessing the epidemiology of diabetes in Canadian children. Pediatr Diabetes 2010; 11(2):122-128.

(10) Antoniou T, Zagorski B, Loutfy MR, Strike C, Glazier RH. Validation of case-finding algorithms derived from administrative data for identifying adults living with human immunodeficiency virus infection. PLoS One 2011; 6(6):e21748.

(11) Tu K, Campbell NR, Chen ZL, Cauch-Dudek KJ, McAlister FA. Accuracy of administrative databases in identifying patients with hypertension. Open Med 2007; 1(1):e18-e26.

(12) Austin PC, Daly PA, Tu JV. A multicenter study of the coding accuracy of hospital discharge administrative data for patients admitted to cardiac care units in Ontario. Am Heart J 2002; 144(2):290-296.

(13) Widdifield J, Bernatsky S, Paterson JM, Tu K, Ng R, Thorne JC et al. Accuracy of Canadian health administrative databases in identifying patients with rheumatoid arthritis: a validation study using the medical records of rheumatologists. Arthritis Care Res (Hoboken) 2013; 65(10):1582- 1591.
(14) Widdifield J, Bombardier C, Bernatsky S, Paterson JM, Green D, Young J et al. An administrative data validation study of the accuracy of algorithms for identifying rheumatoid arthritis: the influence of the reference standard on algorithm performance. BMC Musculoskelet Disord 2014; 15:216.
(15) Robles SC, Marrett LD, Clarke EA, Risch HA. An application of capture-recapture methods to the estimation of completeness of cancer registration. J Clin Epidemiol 1988; 41(5):495-501.
(16) Hall S, Schulze K, Groome P, Mackillop W, Holowaty E. Using cancer registry data for survival studies: the example of the Ontario Cancer Registry. J Clin Epidemiol 2006; 59(1):67-76.

Supplement Table 3. Patient CR Eligible Diagnoses & Source

**Concept**	**Data Sources/ Code Type**	**Algorithm Details**	**Notes**
Myocardial Infarction (MI)	LCVIS	Refevent_ACS_MI = 1	Where r_intake_date is a valid date
Percutaneous Coronary Intervention (PCI)	LCVIS	refevent_PTCA = 1	Where r_intake_date is a valid date
Coronary Artery Bypass Graft Surgery (CABS)	LCVIS	refevent_CABG = 1	Where r_intake_date is a valid date
Unstable Angina (UA)	LCVIS	refevent_ACS__unstable_angina = 1	Where r_intake_date is a valid date
Myocardial Infarction (MI)	Screening database	Reason_for_hosp_admission = 1	Where ‘valid screening date (date_screened) present
Percutaneous Coronary Intervention (PCI)	Screening database	Reason_for_hosp_admission = 3	Where ‘valid screening date (date_screened) present
Coronary Artery Bypass Graft Surgery (CABS)	Screening database	Reason_for_hosp_admission = 4	Where ‘valid screening date (date_screened) present
Unstable Angina (UA)	Screening database	Reason_for_hosp_admission = 2	Where ‘valid screening date (date_screened) present

Supplement Table 4 Administrative Data Codes Used to Define Baseline Characteristics & Source

Characteristic	Data Sources/ Code Type	Code/ Algorithm Details	Notes
Age	RPDB		
Sex	RPDB		
Income quintile	RPDB		
Rurality (rural vs. urban)	RPDB		
Year of cohort entry	Screening database<br>DAD	date_screened	
Time between cohort entry and index date	LCVIS<br>Screening database<br>DAD	r_referral_date<br>date_screened	
Heart Failure	CHF database	-	Prevalent *before* admission for cardiac event
MI	DAD / ICD-9,10	ICD 9: 410, I20	MI *before* admission for cardiac event
PCI	DAD / CCP **or** OHIP Fee	CCP 48.02, 48.03<br>OHIP Z434, G262, G298	PCI *before* admission for cardiac event
CABG	DAD / CCP, CCI **or** OHIP Fee	CCP 48.04, 48.12- 7, 48.19, 48.2-3<br>OHIP E652, R742-3, E654 E645	CABS *before* admission for cardiac event
Unstable Angina	DAD / ICD-9,10	ICD 9: 4130, 4139<br>ICD 10: I200, I2382	UA *before* admission for cardiac event
Atrial fibrillation/flutter	DAD / ICD-9,10	ICD 9: 4273<br>ICD 10: I48	*before* admission for cardiac event
Hypertension	HYPERTENSION	-	Prevalent prior to admission date
Hyperlipidemia	DAD / ICD-9,10	ICD-9: 2722, 2724<br>ICD-10: E782, E784-5	*before* admission for cardiac event
Haemorrhagic stroke	DAD / ICD-9,10	ICD-9: 430-2<br>ICD-10: I62, I64, I600-7, I609, I61	*before* admission for cardiac event
Ischemic stroke	DAD / ICD-9,10<br>NACRS	ICD-9: 4340-1, 4349, 436, 3623<br>ICD-10: I630-5	*before* admission for cardiac event

		I638-9, H340-1	
TIA	DAD / ICD-9,10<br>NACRS	ICD-9: 435<br>ICD-10: H34.0, G45.0-3, G45.8-9	*before* admission for cardiac event
CKD	DAD / ICD-9,10<br>OHIP<br>NACRS	ICD-9: 4030-1, 4039-41, 4049, 585-6, 5888-9, 2504<br>ICD-10: E102, E112, E132, E142, I12, I13, N08, N18, N19	*before* admission for cardiac event
Diabetes mellitus	ODD	-	Prevalent prior to admission date
Peripheral vascular disease	DAD / ICD-9,10	ICD-9: 4402, 4408-9, 5571, 4439, 444<br>ICD-10: I700, I702, I708-9, I709, I731, I738-9, K551	*before* admission for cardiac event
Chronic lung disease (including COPD)	DAD / ICD-9,10<br>OHIP<br>NACRS	ICD-9: 491-6, 500-5, 5064, 5069, 5081, 515-7, 5185, 5188, 5198-9, 4168-9<br>ICD-10: I272, I278-9, J40-5, J47, J60-8, J701, J703-4, J708-9, J82, J84, J92, J941, J949, J953, J961, J969, J984, J988, J989, J99<br>OHIP: J889, J689	*before* admission for cardiac event
Major Cancers	DAD / ICD-9,10<br>OHIP	(on request)	*before* admission for cardiac event<br>Includes: lung/bronchi, colon/rectum, breast, pancreas, prostate, leukeumia, non-Hodgkin lymphoma, liver, ovarian, esophageal
Alcoholism	DAD / ICD-9,10<br>NACRS	ICD-9: 303, 3050<br>ICD-10: E244, E512, F10, G312, G621, G721, I426, K292, K70, K860, T510, X45, X65, Y15, Y573,	*before* admission for cardiac event

		Z502, Z714, Z721	
Obesity	DAD / ICD-9,10 OHIP	ICD-9: 278<br>ICD-10: E660, E662, E668-9	*before* admission for cardiac event
Charlson comorbidity score [4] [5] [6]	DAD / ICD-9,10	(Categorize as: 0-1, 2, 3+, No hospitalizations)	Use all diagnoses; *before* admission for cardiac event
Hospital Episodes	DAD		Count unique EPI variables *before* admission for cardiac event (DO NOT INCLUDE index cardiac hospitalization)
Cardiologist Visit	OHIP	Use FEESUFF="A" & OHIP SPEC=60<br>Restrict to 1 Feecode per physnum per IKN per day	*before* admission for cardiac event
Internal Medicine Visit	OHIP	Use FEESUFF="A" & OHIP SPEC=13<br>Restrict to 1 Feecode per physnum per IKN per day	*before* admission for cardiac event

[4] Charlson, ME, Pompei P, Alex KL, Mackenzie CR: A new method for classifying prognostic comorbidity in longitudinal studies: development and validation. J Chron Dis 1987, 40: 373-383.

[5] Quan H, Sundararajan V, Halfon P, Fong A, Burnand B, Luthi JC, Saunders LD, Beck CA, Feasby TE, Ghali WA. Coding algorithms for defining comorbidities in ICD-9-CM and ICD-10 administrative data. Med Care. 2005 Nov;43(11):1130-9.

[6] Sundararajan V, Henderson T, Perry C, Muggivan A, Quan H, Ghali WA. New ICD-10 version of the Charlson comorbidity index predicted in-hospital mortality. J Clin Epidemiol. 2004 Dec;57(12):1288-94.

Supplement Table 5. Administrative data codes used to define outcomes

Concept	Data Sources/ Code Type	Code/ Algorithm Details	Notes
**COMPOSITE OF:**			Determine time-to-event in days
MI	DAD / ICD-10	As defined in Table 4	
HF	DAD / ICD-10	As defined in Table 4	New admission
PCI	DAD/CCI **or** OHIP Fee	As defined in Table 4	Determine the index date from DAD for PCI. Note that PCI within 6 months of the **cohort entry date** is not considered an outcome
CABG	DAD/CCI **or** OHIP Fee	As defined in Table 4	Determine the index date from DAD for CABG. Note that CABG within 6 months of the **cohort entry date** is not considered an outcome
Death	RPDB	As defined in Table 4	

Supplement Table 6: Pre-match Baseline Patient Characteristics

	Non-CR participants (N=1,192)	CR participants (N=358)	Total (N=1,550)	Standardized difference [7]
**Demographics**				
Age				
Mean (SD)	64.21 ± 11.14	58.80 ± 10.61	62.96 ± 11.25	0.5
Female, N (%)	356 (29.9%)	100 (27.9%)	456 (29.4%)	0.04
**Income quintile**, N (%)				
Quintile 1	<=210	<=65	270 (17.4%)	0.01
Quintile 2	273 (22.9%)	72 (20.1%)	345 (22.3%)	0.07
Quintile 3	220 (18.5%)	71 (19.8%)	291 (18.8%)	0.03
Quintile 4	253 (21.2%)	59 (16.5%)	312 (20.1%)	0.12
Quintile 5	237 (19.9%)	95 (26.5%)	332 (21.4%)	0.16
Missing [8]	<=5	<=5	7	
**Rural**, Yes, N (%)	313 (26.3%)	28 (7.8%)	341 (22.0%)	0.51
**Year of cohort entry**, N (%)				
2002				
2003	147 (12.3%)	28 (7.8%)	175 (11.3%)	0.15
2004	673 (56.5%)	144 (40.2%)	817 (52.7%)	0.33
2005	<=375	<=110	477 (30.8%)	0.03
2006	<=5	<=85	81 (5.2%)	0.75
2007				
2008				
**Index Cardiac Event**, N (%)				
Myocardial Infarction	176 (14.8%)	64 (17.9%)	240 (15.5%)	0.08
Unstable Angina	222 (18.6%)	21 (5.9%)	243 (15.7%)	0.4
Percutaneous coronary intervention	350 (29.4%)	163 (45.5%)	513 (33.1%)	0.34
Coronary artery bypass graft surgery	444 (37.2%)	110 (30.7%)	554 (35.7%)	0.14
**Prior Cardiac Events in the previous 5 years**, N (%)				
Myocardial Infarction	491 (41.2%)	23 (6.4%)	514 (33.2%)	0.89
Unstable Angina	373 (31.3%)	22 (6.1%)	395 (25.5%)	0.68
Percutaneous coronary intervention	101 (8.5%)	12 (3.4%)	113 (7.3%)	0.22
Coronary artery bypass graft surgery	34 (2.9%)	0 (0.0%)	34 (2.2%)	0.24
Heart Failure	190 (15.9%)	10 (2.8%)	200 (12.9%)	0.46

[7] Standardized difference where meaningful difference is greater than 0.1

[8] In the analysis, the missing of income quintile is re-coded as quintile 3

**Comorbidities in the previous 5 years**, N (%)				
Atrial fibrillation/flutter	98 (8.2%)	6 (1.7%)	104 (6.7%)	0.31
Hypertension	288 (24.2%)	78 (21.8%)	366 (23.6%)	0.06
Hyperlipidemia	153 (12.8%)	6 (1.7%)	159 (10.3%)	0.44
Haemorrhagic stroke	<=10	<=5	8 (0.5%)	0.01
Ischemic stroke	<=20	<=5	19 (1.2%)	0.13
Transient Ischemic Stroke	<=25	<=5	22 (1.4%)	0.15
Chronic kidney disease	121 (10.2%)	17 (4.7%)	138 (8.9%)	0.21
Diabetes mellitus	104 (8.7%)	26 (7.3%)	130 (8.4%)	0.05
Peripheral vascular disease	<=45	<=5	45 (2.9%)	0.13
Chronic lung disease (including COPD)	341 (28.6%)	60 (16.8%)	401 (25.9%)	0.29
Major Cancers	93 (7.8%)	29 (8.1%)	122 (7.9%)	0.01
Alcoholism	<=20	<=5	20 (1.3%)	0.14
Obesity	<=45	<=5	49 (3.2%)	0.15
**Charlson Comorbidity Index** [9] [10] [11]				
0,1	595 (49.9%)	78 (21.8%)	673 (43.4%)	0.61
2	183 (15.4%)	15 (4.2%)	198 (12.8%)	0.38
3+	170 (14.3%)	14 (3.9%)	184 (11.9%)	0.37
No Hospitalizations	244 (20.5%)	251 (70.1%)	495 (31.9%)	1.15
**Healthcare system utilization**, N (%)				
Hospital Episodes				
0	244 (20.5%)	251 (70.1%)	495 (31.9%)	1.15
1-5	<=880	<=110	980 (63.2%)	0.98
6+	<=75	<=5	75 (4.8%)	0.31
Visits to a Cardiologist				
0	404 (33.9%)	236 (65.9%)	640 (41.3%)	0.68
1+	788 (66.1%)	122 (34.1%)	910 (58.7%)	0.68
Visits to an Internist				
0	107 (9.0%)	147 (41.1%)	254 (16.4%)	0.8
1-5	226 (19.0%)	104 (29.1%)	330 (21.3%)	0.24
6+	859 (72.1%)	107 (29.9%)	966 (62.3%)	0.93

[9] Charlson, ME, Pompei P, Alex KL, Mackenzie CR: A new method for classifying prognostic comorbidity in longitudinal studies: development and validation. J Chron Dis 1987, 40: 373-383.

[10] Quan H, Sundararajan V, Halfon P, Fong A, Burnand B, Luthi JC, Saunders LD, Beck CA, Feasby TE, Ghali WA. Coding algorithms for defining comorbidities in ICD-9-CM and ICD-10 administrative data. Med Care. 2005 Nov;43(11):1130-9.

[11] Sundararajan V, Henderson T, Perry C, Muggivan A, Quan H, Ghali WA. New ICD-10 version of the Charlson comorbidity index predicted in-hospital mortality. J Clin Epidemiol. 2004 Dec;57(12):1288-94.
